# Early intervention with tafamidis provides long-term benefit in delaying neurological progression in patients with transthyretin familial amyloid polyneuropathy

**DOI:** 10.1186/1750-1172-10-S1-P12

**Published:** 2015-11-02

**Authors:** Marcia Waddington-Cruz, Leslie Amass, Denis Keohane, Jeffrey Schwartz, Balarama Gundapaneni, Hui Hua Li

**Affiliations:** 1 Hospital Universitario Clementino, Neuromuscular Diseases Unit, 21941-913, Rio de Janeiro, Brazil; 2 Pfizer, Pfizer, PA 19426, Collegeville, USA; 3 Pfizer, Pfizer, NY 10017, New York, USA; 4 Pfizer, Pfizer, CT 06340, Groton, USA; 5 InVentiv Health, Statistics, MA 01803, Burlington, USA

## Background

Tafamidis is a transthyretin (TTR) stabilizer approved to delay neurological progression associated with stage 1 TTR familial amyloid polyneuropathy (FAP). A placebo-controlled, randomized 18-month registration trial allowed for continued evaluation of patients receiving tafamidis (20 mg oral once-daily) through an ongoing open label extension study. The effectiveness of tafamidis for delaying long-term neurological progression relative to baseline levels of neuropathy impairment at the start of treatment has not been reported previously.

## Methods

This analysis describes the trajectory of the disease progression over 5.5 years or more for 71 patients with the V30M mutation and stage 1 TTR-FAP who received tafamidis either at the start of the registration trial or after the switch from placebo following 18 months of study participation. All V30M patients treated with tafamidis in the original trial or its extension and for whom follow-up data were available were included. The impact of tafamidis on neurological progression over time was evaluated using the Neuropathy Impairment Scale for lower limbs (NIS-LL). Baseline NIS-LL at active treatment start was defined as the last measurement before the first active dose of tafamidis.

## Results

Patients (30 male, 41 female) were primarily Caucasian (91.5%) with a mean (standard deviation [SD]) age of disease onset of 35.7 (11.31) years. Very low baseline disease severity, defined as NIS-LL ≤10 at treatment start (baseline mean NIS-LL [SD] = 4.1 [3.1]), was associated with a minimal level of disease progression over time. Mean change in NIS-LL from baseline (SD) over 5.5 years of treatment was: 0.6 (3.2) at 1 year, 1.3 (3.7) at 2 years, 2.3 (6.0) at 3 years, 1.7 (4.8) at 4 years and 5.3 (10.1) at 5.5 years. Additional categorization of patients according to baseline NIS-LL of 0-5 and 5-10 revealed a similar pattern of reduced disease trajectory. For 3 patients, there was evidence after 3-5 years of treatment of an increase in the NIS-LL score to 30 or more.

## Conclusion

Early intervention with tafamidis provides long-term benefit in delaying neurological progression associated with TTR-FAP. These data underscore the need to intervene as early as possible with symptomatic TTR-FAP patients.

## Note

First presented at the Peripheral Nerve Society Congress, Montreal, Canada, June 2015.

